# Parenting practices and interventions during the COVID-19 pandemic lockdown: an exploratory cross-sectional study of caregivers in Brazil, Mexico, and the United States

**DOI:** 10.1186/s41155-024-00295-1

**Published:** 2024-04-07

**Authors:** Mariana M. Juras, Acileide Cristiane F. Coelho, Alejandro L. Vázquez, Michela Ribeiro, Marina Kohlsdorf, Alice Lima Custódio, Nancy G. Amador Buenabad, Lucia Vazquez Perez, Cole Hooley, Miya L. Barnett, Ana A. Baumann

**Affiliations:** 1 Florida Institute of Technology, Melbourne, USA; 2 Universidade de Brasília, Brasília, Brazil; 3 The University of Tennessee, Knoxville, Knoxville, USA; 4 Aiutare Instituto de Psicologia, Brasília, Brazil; 5 Sam Houston State University, Huntsville, USA; 6 National Institute of Psychiatry Ramon de la Fuente Muniz, Huntsville, Mexico; 7 Brigham Young University, Provo, USA; 8 University of California, Santa Barbara, USA; 9 Washington University in St. Louis, St. Louis, USA

**Keywords:** COVID-19 pandemic, Parenting, Cross-cultural comparison, Telehealth

## Abstract

**Introduction:**

The COVID-19 pandemic led countries’ governments to rapidly establish lockdowns and social distancing, which altered family routines and the quality of family relationships worldwide.

**Objectives:**

This exploratory cross-sectional study aimed to identify the impacts of the social distancing and lockdown in parenting practices of caregivers from Brazil, Mexico, and the USA, and to analyze the continuity of parenting intervention support for children and their families at the beginning of the pandemic in these countries.

**Methods:**

The sample consisted of 704 caregivers of children (286 from Brazil, 225 from Mexico, and 193 from the USA) who answered an online survey about parenting practices before/after quarantine, caregiver/child routines, feelings related to quarantine, changes in everyday life since the beginning of the COVID-19 pandemic, contact with health professionals, and sources of parenting information.

**Results:**

Data indicate that caregivers from the three countries experienced similar parenting practices during this time, and did not report significant changes before and after the lockdown. They sought information about parenting predominantly via social media. Those receiving previous mental health care perceived the transition from in-person to telehealth services during the pandemic as feasible and acceptable.

**Conclusion:**

This study will be helpful for clinicians and parents to contextualize their practices amid long-standing effects that the COVID-19 pandemic can have on children and their families during and post-pandemic from multiple cultural backgrounds.

## Introduction

The COVID-19 pandemic led governments to rapidly implement lockdowns and social distancing policies changing family routines globally. Family routines and the quality of family relationships changed (La Iglesia, 2021; Marques et al., 2020; Roos et al., 2021), with several caregivers working remotely from home while taking care of daily family routines and receiving little social support (Roos et al., 2021; Schmidt et al., 2020). This isolation affected families' economic aspects (Avery et al., 2021; Roos et al., 2021) and children's schooling practices and contact with extended family members (Quetsch et al., 2022).

Health and economic uncertainties combined with the lockdown measures during the first phase of the COVID-19 pandemic were associated with an increase in children and families vulnerabilities, such as poverty, domestic violence, and mental health problems (Avery et al., 2021; Campbell, 2020; Roos et al., 2021; Valdez et al., 2020). Families reported an increase in stress, anxiety, depression, and feelings of being overburdened (Adams et al., 2021; Griffith, 2022; Linhares & Enumo, 2020; López Garza et al., 2021; Vescovi et al., 2021). Parents’ strategies to deal with those stressors included establishing a new routine for children, family activities, and remote connections with extended family (Adams et al., 2021).

To continue to provide services, mental health organizations encouraged and supported the transition to telehealth services during the pandemic (Barnett et al., 2021; Schmidt et al., 2020; Schoebel et al., 2021). Broadly, data from the USA indicate that remote services for mental health have similar outcomes to in-person services and that telehealth is acceptable and feasible (Schoebel et al., 2021; Shigekawa et al., 2018; Snoswell et al., 2023). Specifically to parenting interventions, data also indicate that parents of children consider telehealth delivery of interventions acceptable and effective in supporting their parenting practices (de Nocker & Toolan, 2023; Hippman et al., 2023; Pan et al., 2023).

These studies on the feasibility and acceptability of telehealth delivery of parenting practices, however, tend to be specific to one country. With the assumption that, while parenting practices may have some universal components, some parenting techniques may be specific to culture (Maciel et al., 2023; Martinez et al., 2020), it is unclear whether the changes to telehealth services during the pandemic were universally considered appropriate by caregivers across different countries. To address this gap, this study aimed to explore how telehealth for parenting interventions was perceived by caregivers from Brazil, Mexico, and the USA.

Understanding how caregivers perceived changes to telehealth in a cross-country study can be helpful for clinicians to contextualize their services amid the long-standing effects of the COVID-19 pandemic as parenting interventions are being disseminated in different countries (Maciel et al., 2023). These three countries were selected because, combined, they had a high number of deaths due to SARS-CoV-2. In total, they reached around 2.2 million deaths, with the USA and Brazil occupying the first and second positions, respectively, and Mexico in fifth position in the number of deaths (World Health Organization, 2023). These countries were also chosen because they had different intensities in terms of the uptake of the shutdown measures and had variability in terms of individual compliance with the different public health measures during the pandemic (Bennouna et al., 2021; Testa et al., 2021). Moreover, we have collaborators in each of the countries either implementing or interested in implementing evidence-based parenting interventions (Baumann et al., 2019; Baumann et al., 2022; Domenech Rodríguez et al., 2018; Parra-Cardona et al., 2021), and therefore, we positioned this study as an exploratory cross-sectional survey to understand how caregivers experience changes in their family routines and access to services during the COVID-19 pandemic to inform future studies.

## Methods

This cross-sectional exploratory study aimed to examine potential differences in parenting practices, routines, and perspectives on using parenting interventions through telehealth among caregivers from Brazil, Mexico, and the USA. This study was approved by the Washington University in St. Louis Institutional Review Board (IRB (Protocol #202004215).

### Data collection

We recruited participants via snowball sampling. Emails were sent to groups of providers from our professional networks, listservs from psychological institutes, and graduate courses (e.g., *Associação Nacional de Pesquisa e Pós Graduação em Psicologia* [Brazilian National Psychological Research and Graduate Programs Association], ANPEEP–https://www.anpepp.org.br/), and we posted notes in Facebook and Twitter in all three languages (Portuguese, Spanish, and English). Up to three emails were sent to our networks. Informal contacts were also made through these organizations using WhatsApp groups. The survey was administered to participants using Qualtrics, an online survey tool, and there were no payments or reimbursements to participants. The English survey collected data from April 29 to October 2, 2020; the Spanish survey from June 4 to October 28, 2020; and the Portuguese survey from May 1 to September 8, 2020.

### Measures

The survey was organized into 8 sections, described below.

#### Demographics

Participants reported the following demographic information: age, gender, history of chronic disease, number of and relation to children, whether their child had a chronic disease, number of individuals within the home, education, marital status, employment status, and household income. Socioeconomic status was categorized into low, medium, and high across countries using guidance from the Pew Research Center (Kochhar, 2021).

#### Parenting practices

Participants completed an adapted version of the Alabama Parenting Questionnaire (Essau et al., 2006), which consisted of 22 items assessing a wide variety of parenting practices, including positive parenting, involvement, inconsistent discipline, poor monitoring/supervision, corporal punishment, and other discipline practices, such as the use of time out. Participants were asked to report the extent to which they used specific parenting practices before and after the start of the COVID-19 pandemic. Responses included *never* (1), *almost never* (2), *sometimes* (3), *often* (4), and *always* (5). Mean scores were calculated for pre- and post-pandemic parenting practices. Internal consistency of the APQ in this study was poor within the overall sample (Cronbach’s alpha pre = .485, post = .539). Considering bias in reporting punishment practices against children, dichotomous indicators were created to represent whether participants *used* (1; almost never to always) or *did not use* (0; never) specific parenting practices during the COVID-19 pandemic.

#### Caregiver activities and routines

Participants were asked to report whether or not they were engaging in a list of activities with their child(ren) across 11 activities on a survey developed by the research team. The activities included: house cleaning and organizing, cooking and baking, school work, games, and fun activities, searching for information about COVID-19 and its repercussions, caring for other family members, remote contact with other family members, caring for pets, physical activities, religious activities, and other.

#### Feelings related to COVID-19 quarantine

Participants were provided with five emotions: calm, hopeful, irritated, fearful, and tired. They were asked to rate their intensity on a scale from 1 to 10, where lower scores indicated less intensity and higher scores indicated greater intensity.

#### Changes in everyday life

Participants completed the Epidemic–Pandemic Impact Inventory (EPII) (Grasso et al., 2020). This measure includes 90 statements that indicate potential shifts that may have occurred during the pandemic for the caregiver across several domains, including work, home, social functioning, health, and positive change (e.g., “more quality time with family”). For each statement, participants would select if the change occurred and for whom (*yes (me)* [1], *yes (person in home)* [2], *no* [3]). Responses were recoded as *yes* (1) or *no* (0).

#### Contact with health professionals

Participants were asked how they were connecting with health professionals (online [e.g., Zoom, Webex], phone, Instagram, Facebook, or other). They also completed three yes (1) or no (0) items assessing whether this method of contact was new and whether they felt that the professional acknowledged them and their child as a person.

#### Sources of parenting information

Participants were asked to report where they accessed information on parenting during the pandemic. We provided 11 sources of parenting information (e.g., social media, news, TV, books, health providers) and they were asked to select all sources they sought information from.

### Translation process of surveys

The English version of EPII (Grasso et al., 2020) and APQ scales were translated by NAB into Mexican Spanish and by ALC into Brazilian Portuguese and checked for accuracy by AB. The other measures were originally created by ALC, MR, and ACC in Brazilian Portuguese, and were translated by ALC into English and by NAB into Mexican Spanish. The translated versions were then back-translated by MR and ACC into Brazilian Portuguese. AB supervised and checked all translations for accuracy and the final surveys were approved by all authors. We did not conduct cognitive interviews because of the urgency in collecting the data considering the fast pace of the effects of the pandemic on parenting practices.

### Analysis

Across all three surveys, 19.4% of data was missing. Information from all available responses was used. Data were analyzed using descriptive statistics depending on whether variables were categorical (i.e., chi-square test of independence) or continuous (i.e., Kruskal-Wallis rank sum test) to examine potential differences in parenting practices and feelings between countries.

## Results

### Participants’ demographics

Participants of this study consisted of 704 caregivers of children (ranging from infancy to 17 years of age) from Brazil, Mexico, and the USA. The majority of the sample were mothers (85.5%), married or lived in cohabitation (74.9%), and from upper-middle- and high-income socioeconomic status (66.8%). In terms of geographic location, the American sample was mostly distributed in the Midwest (Missouri 55%; Illinois 15%), the Brazilian sample was mostly from the Midwest (Distrito Federal 57%; Goias 5%), and the Southeast (Sao Paulo 22%; Rio de Janeiro 7%), and Mexican sample mostly lived in the Central region (Mexico State 70%) (Table 1).

**Table 1:** Demographic characteristics of the sample

Mean/count (SD/%)	Total	Brazil	Mexico	USA
	*n* = 704	*n* = 286	*n* = 225	*n* = 193
Caregiver age	41.0 (8.2)	42.0 (7.6)	38.8 (8.2)	41.9 (8.6)
Caregiver gender				
Female	640 (90.9%)	254 (88.8%)	206 (91.6%)	180 (93.3%)
Male	59 (8.4%)	31 (10.8%)	16 (7.1%)	12 (6.2%)
Caregiver chronic disease				
Yes	151 (21.4%)	65 (22.7%)	41 (18.2%)	45 (23.3%)
No	544 (77.3%)	217 (75.9%)	181 (80.4%)	146 (75.6%)
Number of children	1.8 (1.2)	1.7 (1.0)	1.7 (1.4)	1.9 (1.0)
Relation to children				
Mother	602 (85.5%)	244 (85.3%)	184 (81.8%)	174 (90.2%)
Father	48 (6.8%)	25 (8.7%)	12 (5.3%)	11 (5.7%)
Stepmother	1 (0.1%)	0 (0%)	0 (0%)	1 (0.5%)
Stepfather	2 (0.3%)	1 (0.3%)	0 (0%)	1 (0.5%)
Grandmother	14 (2%)	5 (1.7%)	6 (2.7%)	3 (1.6%)
Grandfather	0 (0%)	0 (0%)	0 (0%)	0 (0%)
Aunt	13 (1.8%)	4 (1.4%)	8 (3.6%)	1 (0.5%)
Uncle	1 (0.1%)	1 (0.3%)	0 (0%)	0 (0%)
Other	12 (1.7%)	3 (1%)	8 (3.6%)	1 (0.5%)
Child chronic disease				
Yes	78 (11.1%)	37 (12.9%)	16 (7.1%)	25 (13%)
No	613 (87.1%)	244 (85.3%)	202 (89.8%)	167 (86.5%)
Number of people in the home	4.0 (1.6)	3.9 (1.6)	4.0 (1.9)	4.0 (1.3)

### Parenting practices

Across the three countries, there were no significant differences in cumulative scores representing discipline practice toward children prior to and during the pandemic (*p* > .05). However, internal consistency for these scales was poor, which suggests that individual items may not represent a single latent factor. Descriptively, there were differences in individual parenting practices between countries. We present individual responses from the APQ during the pandemic to provide fine-grain detail on differences in parenting practices. Overall, positive parenting and involvement items did not vary significantly between countries, with the exception of two items: “volunteer to help with special activities child is involved in” significantly higher (*p* < .001) in Brazil (78%) and lower in Mexico (63.6%) and “rewarding the child for behaving well” was significantly higher (*p* < .001) in the USA (75.6%) and lower in Mexico (53.3%). Inconsistent discipline was a practice generally more common for American participants while corporal punishment was significantly higher in Brazil and Mexico (see Table 2).

**Table 2:** Difference in individual parenting practices by country

	Total	Brazil	Mexico	USA	*p* value*
	*n* = 704	*n* = 286	*n* = 225	*n* = 193	
Volunteer to help with special activities child is involved in					< .001
Yes	498 (70.7%)	223 (78%)	143 (63.6%)	132 (68.4%)	
No	44 (6.2%)	6 (2.1%)	16 (7.1%)	22 (11.4%)	
Reward or give something extra to child for obeying or behaving well					< .001
Yes	441 (62.6%)	175 (61.2%)	120 (53.3%)	146 (75.6%)	
No	87 (12.4%)	53 (18.5%)	27 (12%)	7 (3.6%)	
Child talks parent out of being punished					<.001
Yes	320 (45.5%)	175 (61.2%)	45 (20%)	100 (51.8%)	
No	186 (26.4%)	49 (17.1%)	84 (37.3%)	53 (27.5%)	
Let child out of a punishment early					<.001
Yes	301 (42.8%)	126 (44.1%)	58 (25.8%)	117 (60.6%)	
No	194 (27.6%)	97 (33.9%)	62 (27.6%)	35 (18.1%)	
Parent busy and forgets what child is doing					<.001
Yes	331 (47%)	159 (55.6%)	59 (26.2%)	113 (58.5%)	
No	182 (25.9%)	63 (22%)	81 (36%)	38 (19.7%)	
Child not punished when she/he has done something wrong					<.001
Yes	344 (48.9%)	158 (55.2%)	61 (27.1%)	125 (64.8%)	
No	149 (21.2%)	61 (21.3%)	59 (26.2%)	29 (15%)	
Punishment they give their child depends on their mood					<.001
Yes	358 (50.9%)	163 (57%)	76 (33.8%)	119 (61.7%)	
No	155 (22%)	57 (19.9%)	64 (28.4%)	34 (17.6%)	
Spank child with hand when she/he has done something wrong					0.002
Yes	158 (22.4%)	72 (25.2%)	55 (24.4%)	31 (16.1%)	
No	356 (50.6%)	148 (51.7%)	87 (38.7%)	121 (62.7%)	
Ignore child when she/he is misbehaving					<.001
Yes	292 (41.5%)	135 (47.2%)	50 (22.2%)	107 (55.4%)	
No	226 (32.1%)	86 (30.1%)	94 (41.8%)	46 (23.8%)	
Slap child when she/he has done something wrong					<.001
Yes	100 (14.2%)	75 (26.2%)	13 (5.8%)	12 (6.2%)	
No	430 (61.1%)	148 (51.7%)	141 (62.7%)	141 (73.1%)	
Take away privileges or money from child as punishment					0.001
Yes	396 (56.2%)	159 (55.6%)	106 (47.1%)	131 (67.9%)	
No	130 (18.5%)	59 (20.6%)	49 (21.8%)	22 (11.4%)	
You hit your child with belt, switch, other object when she/he has done something wrong^a^					
Yes	33 (4.7%)	13 (4.5%)	18 (8%)	2 (1%)	
No	499 (70.9%)	208 (72.7%)	140 (62.2%)	151 (78.2%)	
Yell or scream at child when she/he has done something wrong					0.013
Yes	410 (58.2%)	190 (66.4%)	99 (44%)	121 (62.7%)	
No	96 (13.6%)	30 (10.5%)	35 (15.6%)	31 (16.1%)	
Use time out as punishment					0.007
Yes	262 (37.2%)	103 (36%)	64 (28.4%)	95 (49.2%)	
No	249 (35.4%)	112 (39.2%)	78 (34.7%)	59 (30.6%)	
Give child extra chores as punishment					< .001
Yes	235 (33.4%)	74 (25.9%)	74 (32.9%)	87 (45.1%)	
No	278 (39.5%)	144 (50.3%)	68 (30.2%)	66 (34.2%)	

*P values indicate significant differences between countries

^a^Chi-square test not conducted due to cross-tabulation cell sparsity

### Caregiver activities and routines

Participants from the three countries reported similar frequencies of some listed activities in which they were involved with their children. As participants could provide multiple responses, we will describe frequently endorsed activities and routines. The most frequent activities reported during the pandemic were: “games and fun activities” (66.8%), “house cleaning and organization” (66.3%), “remote contact with family and friends” (58.5%), “school work” (57.4%), and “cooking and baking” (54.8%). The least prevalent activities reported by participants from three countries were “search for information about COVID-19 and its repercussions” (17.9%) and “taking care of other family members” (19.7%).

There were some differences in terms of the types of activities reported by each nationality. Participating in physical activities with their children was mostly reported by 61.1% of American participants, compared to 47.6% of Mexicans, and only 36.7% of Brazilians. Most Americans (56.4%) reported taking care of pets, compared to 48.9% of Mexicans and 38.8% of Brazilians. Finally, including children in religious activities or rituals was listed by 30.1% of Americans, 24.5% of Brazilians, and only 10.7% of Mexicans.

### Feelings regarding COVID-19 quarantine

When questioned about their feelings regarding the social distancing measures, feeling tired was the highest rate reported by participants across all three countries. Furthermore, Brazilian participants rated feeling tired significantly higher than their counterparts from Mexico and the USA (*p* < 0.05). Feeling irritated and fearful received the lowest participants’ scores, with American participants’ rating of feeling fearful significantly lower (*p* < 0.001; see Table 3). In general, all participants also reported feeling hopeful (6.1) and calm (5.9) (scale of 0–10).

**Table 3:** Caregiver feelings regarding COVID-19 quarantine

	Total	Brazil	Mexico	USA	*p* value
	*n* = 704	*n* = 286	*n* = 225	*n* = 193	
Calm	5.9 (2.3)	6.0 (2.2)	6.1 (2.4)	5.6 (2.4)	>.05
Hopeful	6.1 (2.6)	6.5 (2.6)	6.3 (2.7)	5.5 (2.5)	.002
Irritated	5.3 (2.6)	5.5 (2.6)	4.9 (2.6)	5.5 (2.6)	.05
Fearful	5.4 (2.8)	5.8 (2.9)	5.5 (2.8)	4.6 (2.7)	<.001
Tired	6.8 (2.9)	7.3 (2.7)	6.4 (3.1)	6.5 (2.9)	.01

Subjective units of distress: *1* indicates less intensity and *10* indicates great intensity. *p* values represent the Kruskal-Wallis rank sum test

### Changes in everyday life after COVID-19

From the results of the EPII, participants from the three countries and their families were overall impacted similarly by the COVID-19 pandemic. Due to a high degree of missing data, the overall work, home, social, health, and positive impact of the COVID-19 pandemic will not be reported, and the analysis will focus on individual items for the other 5 subscales.

#### Impact of COVID on work

When inquired about the impacts of the COVID pandemic on their work, the majority of the respondents from the three countries reported that the adults in their households were able to maintain their jobs. However, a significantly higher number of participants from Mexico (19.1%) were laid off from their jobs compared to Americans (10.4%) and Brazilians (9.4%). Moreover, 36.4% of participants from Brazil and Mexico or someone in their homes had to reduce their work hours or were furloughed compared to only 21.2% of Americans. A lot more Brazilian participants reported firing or furloughing people they supervised (15.4%), compared to 9.3% of Mexican and 4.7% of American participants. Because of the pandemic, 49.3% of participants also reported feeling less efficient or productive in work, employment, or school compared to 17.2% who reported feeling more efficient. Furthermore, 39.5% of participants reported having a hard time doing their jobs well because of needing to take care of people in the home, compared with 22.2% that did not have this experience.

#### Impact of COVID on their home

Most respondents (45.9%) reported that they had to take over teaching or instructing responsibilities for their children. Participants reported less availability of childcare or babysitting services when needed in the USA compared to Mexico and Brazil. One quarter (26.8%) of participants recognized having more conflict with children or harsher disciplining them. The majority of respondents did not notice any increases in verbal or physical family arguments or conflicts during the first wave of the pandemic. However, for the participants who did notice an increase, the conflicts were more related to verbal arguments or conflict with their spouses or partners (21.4%) and with other adults in the house (12.4%).

#### Impact of COVID on social functioning

There were no significant differences between countries regarding interaction with extended family and friends. Overall, 58.9% of participants reported they had to be separated from family or close friends and 63.9% had their family celebrations canceled or restricted. Moreover, 55.3% of participants also reported they were unable to do enjoyable activities or hobbies, compared to 11.8% of participants that were able to continue these activities.

#### Impact of COVID on emotional health and well-being

Approximately one-third of participants reported an increase in child behavioral or emotional problems as well as in the child’s sleep difficulties or nightmares. Nearly half of the participants reported increased mental health problems or symptoms for themselves, such as mood, anxiety, and stress (see Table 4). The majority of participants did not notice any changes in the use of alcohol and other drugs during the lockdown.

**Table 4:** Increase in mental health problems or symptoms

	Total	Brazil	Mexico	USA
	*n* = 704	*n* = 286	*n* = 225	*n* = 193
Increase in child behavioral or emotional problems				
No	222 (31.5%)	97 (33.9%)	64 (28.4%)	61 (31.6%)
Yes	249 (35.4%)	95 (33.2%)	78 (34.7%)	76 (39.4%)
Increase in child’s sleep difficulties or nightmares				
No	271 (38.5%)	122 (42.7%)	69 (30.7%)	80 (41.5%)
Yes	197 (28%)	68 (23.8%)	72 (32%)	57 (29.5%)
Increase in mental health problems or symptoms (e.g., mood, anxiety, stress)				
No	131 (18.6%)	48 (16.8%)	43 (19.1%)	40 (20.7%)
Yes	344 (48.9%)	147 (51.4%)	100 (44.4%)	97 (50.3%)
Increase in sleep problems or poor sleep quality				
No	135 (19.2%)	53 (18.5%)	34 (15.1%)	48 (24.9%)
Yes	334 (47.4%)	138 (48.3%)	108 (48%)	88 (45.6%)
Increase in use of alcohol or substances				
No	319 (45.3%)	121 (42.3%)	101 (44.9%)	97 (50.3%)
Yes	119 (16.9%)	61 (21.3%)	21 (9.3%)	37 (19.2%)

Yes and no answers are reported to maintain consistency with previous data-informed, considering the missing values for accurate interpretation

#### Impact of COVID on physical health

The majority of respondents from the three countries reported an increase in unhealthy habits, such as sedentarism and overeating, after the pandemic started (see Table 5).

**Table 5:** Increase in unhealthy habits

	Total	Brazil	Mexico	USA
	*n* = 704	*n* = 286	*n* = 225	*n* = 193
Spent more time on screens and devices (e.g., looking at phone, playing video games, watching TV)				
No	55 (7.8%)	25 (8.7%)	18 (8%)	12 (6.2%)
Yes	418 (59.4%)	169 (59.1%)	123 (54.7%)	126 (65.3%)
Less physical activity or exercise.				
No	96 (13.6%)	19 (6.6%)	30 (13.3%)	47 (24.4%)
Yes	369 (52.4%)	167 (58.4%)	112 (49.8%)	90 (46.6%)
More time sitting down or being sedentary				
No	68 (9.7%)	28 (9.8%)	18 (8%)	22 (11.4%)
Yes	406 (57.7%)	168 (58.7%)	124 (55.1%)	114 (59.1%)
Overeating or eating more unhealthy foods (e.g., junk food)				
No	154 (21.9%)	56 (19.6%)	56 (24.9%)	42 (21.8%)
Yes	318 (45.2%)	139 (48.6%)	86 (38.2%)	93 (48.2%)

Yes and no answers are reported to maintain consistency with previous data-informed, considering the missing values for accurate interpretation

#### Positive impact of COVID

Regarding perceptions of quality of family time during the pandemic, 59.7% of participants from the three countries reported having more quality time with their children, and 37.8% perceived higher quality time with their partners or spouses. Moreover, 48.7% also reported more quality time with family or friends in person or from a distance (e.g., on the phone, email, and social media).

### Contact with health professionals

Table 6 shows participants’ perceptions of telehealth services. Only one-quarter of participants (26.5%) informed that they continued to receive services from health professionals after the pandemic started, from which the majority received telehealth services: 15.3% online video-conferencing (e.g., Zoom, Webex) and 7% via phone calls. Overall, they agreed that this new method seems possible, suitable, and doable, but were overall neutral regarding their preference and appeal towards this modality. Most respondents reported feeling like their provider knew them and their children as a person.

**Table 6:** Continued services with health professionals

	Total	Brazil	Mexico	USA
	*n* = 704	*n* = 286	*n* = 225	*n* = 193
Online (e.g., Zoom, Webex)	108 (15.3%)	39 (13.6%)	50 (22.2%)	19 (9.8%)
By phone	49 (7%)	5 (1.7%)	35 (15.6%)	9 (4.7%)
Instagram	2 (0.3%)	1 (0.3%)	1 (0.4%)	0 (0%)
Facebook	13 (1.8%)	1 (0.3%)	10 (4.4%)	2 (1%)
Other	15 (2.1%)	2 (0.7%)	9 (4%)	4 (2.1%)
Different methods of meeting				
Yes	113 (16.1%)	39 (13.6%)	54 (24%)	20 (10.4%)
No	26 (3.7%)	4 (1.4%)	18 (8%)	4 (2.1%)
Feel professional knew them as a person				
Yes	117 (16.6%)	38 (13.3%)	58 (25.8%)	21 (10.9%)
No	17 (2.4%)	7 (2.4%)	8 (3.6%)	2 (1%)
Feel professional knew child as a person				
Yes	110 (15.6%)	35 (12.2%)	54 (24%)	21 (10.9%)
No	28 (4%)	10 (3.5%)	15 (6.7%)	3 (1.6%)

Frequency is reported considering the entire sample to maintain consistency with previous data-informed, considering the missing values for accurate interpretation

### Sources of parenting information

About 2/3 of the participants (63.1%) reported that they sought additional information about parenting and child-rearing during the pandemic. American participants were the least likely to report seeking additional information (45.1%). The sources of access to information varied. Mexicans most significantly sought information via Facebook, healthcare professionals, and books. Americans primarily turned to Facebook, the news, and healthcare professionals. Brazilians accessed Instagram, health professionals, and the news.

## Discussion

This cross-sectional exploratory study examined potential differences in parenting practices and routines during the COVID-19 pandemic lockdown from Brazilian, Mexican, and American caregivers and their perceptions of using parenting interventions through telehealth. Results indicated that participants experienced similar child-caregiver routines during this period and did not report significant changes in parenting practices before and after the lockdown. These similarities across countries could be due to the homogeneity of this study’s sample, which consisted predominantly of mothers living in urban areas with a partner from middle socioeconomic status and above. Upper socioeconomic classes, with privileged access to resources, were the least impacted socially, economically, and health-wise by the COVID-19 pandemic (Bambra et al., 2020; Ben Brik et al., 2022; Valenzuela et al., 2020).

While most participants did not significantly report increased levels of family conflict, it is noticeable that nearly one-third of participants reported increased conflict with their partners and other adults in the house, and one-quarter recognized having more conflict with children or harsher disciplining practices according to EPII items. Our data is similar to other reports showing an increase in caregiving stress, intrapersonal conflict, and an increase in harsh parenting during the COVID-19 pandemic (Essler et al., 2023; Feinberg et al., 2022; Fosco et al., 2022). There were differences in parenting practices across countries: inconsistent discipline was more commonly reported by American participants, while corporal punishment was significantly higher in Brazil and Mexico. Differences in parenting practices across cultures in our study are similar to other studies, indicating that while there are some common practices in caregivers across different countries, practices such as discipline and types of attachment may be permeated by cultural context (Cuartas et al., 2019; Hill et al., 2003; Varela et al., 2004). The data from this and other studies will be important to consider as treatment developers adapt parenting interventions for different cultures as we consider the potential long-term mental health outcomes as a consequence of the pandemic (Campbell, 2020; López Garza et al., 2021; Newlove-Delgado et al., 2023; Roos et al., 2021).

When asked about participants’ perceptions about telehealth, they reported these services as feasible and acceptable. These data are similar to other findings from American (de Nocker & Toolan, 2023; Hippman et al., 2023; Schoebel et al., 2021; Shigekawa et al., 2018) and Latin American samples (Phenicie et al., 2021; Santos et al., 2023; Silva et al., 2020; Vázquez et al., 2021) and inform that remote services for mental health seem to be a viable way to address mental health issues for these populations. However, expanding empirical studies, particularly with Brazilian and Mexican samples, is still needed. Furthermore, investing in adequate training for professionals and caregivers in digital inclusion to provide universal access to remote services is equally important, especially for vulnerable populations that need them most (Coelho & Conceição, 2021; Silva et al., 2020). Socio-digital inclusion is also crucial to encourage the representation of these populations in research carried out via digital means because socioeconomically vulnerable and digitally excluded populations may have more difficulty accessing remote research.

We also asked participants where they searched for information about parenting practices and the pandemic. Overwhelmingly, they reported seeking information about parenting predominantly via social media. Our data and those of others indicate the importance of expanding and adapting evidence-based parenting practices via social media to increase the reach of accurate information about evidence-based parenting practices (Schoebel et al., 2021; Vázquez et al., 2021).

## Limitations

This study has limitations. Even though participants were from different countries, the sample was predominantly comprised of mothers from a middle to upper-class background. The nature (online), timing (during lockdown) of the recruitment, and study method (online survey) affected the reach of our sample during a period when our research team did not have resources or were allowed to engage with in-person recruitment for research. Our data, therefore, needs to be interpreted with caution as it does not represent caregivers of diverse economic status within the three countries. Data from other studies indicate that parents from lower socio-economic status faced higher challenges during the pandemic regarding their parenting practices (Kerr et al., 2021). The data from our study and others show the importance of supporting all parents in their practice to improve parenting practices and child outcomes.

The data collection timeframe was also limited during the first pandemic wave; a follow-up cohort could inform the impact of the other SARS-COVID-19 pandemic on parenting practices. Even though these countries started lockdown procedures at similar times (i.e., March 2020), openings progressed differently in each country and region, which may have impacted results. Considering that results relied on self-report, there may have been an increase in response bias. Finally, the internal consistency of measures was poor, and results must be interpreted with caution.

## Conclusion

This exploratory study shows that children’s caregivers from Brazil, Mexico, and the USA did not perceive a significant change in parenting practices before and after the lockdown. However, participants reported an increase in unhealthy habits and mental health issues during lockdown. These impacts from the COVID-19 pandemic are relevant to inform clinical work with children and caregivers in the post-pandemic. This study also provides culturally sensitive information about corporal punishment of children disclosed significantly more by the Latin American sample. These results have clinical implications important to consider when working with caregivers from different countries.

This study also highlights the importance of social media as one of the main sources of information for caregivers in these countries. Scholars interested in disseminating information about evidence-based parenting interventions should consider social media as a crucial source of information for children's caregivers. Finally, our data show that telehealth services were considered feasible/acceptable by children’s caregivers. It is important to acknowledge the pandemic’s influence on the use of technology when adapting evidence-based interventions for Brazilian, Mexican, and American caregivers. Socio-digital inclusion is crucial to increase the accessibility of remote parenting intervention designs and foster these populations’ representation in future studies.

## Data Availability

The datasets used and/or analyzed during the current study are available from the corresponding author upon reasonable request.

## Abbreviations

ANPEEP: *Associação Nacional de Pesquisa e Pós Graduação em Psicologia* [Brazilian National Psychological Research and Graduate Programs Association]
APQ: Alabama parenting questionnaire
COVID-19: Coronavirus disease
EPII: Epidemic–pandemic impact inventory
IRB: Institutional review board
SARS-CoV-2: Severe acute respiratory syndrome coronavirus 2
USA: United states of america
TV: Television
